# Hepatic, Muscle and Intestinal Oxidative Status and Plasmatic Parameters of Greater Amberjack (*Seriola dumerili*, Risso, 1810) Fed Diets with Fish Oil Replacement and Probiotic Addition

**DOI:** 10.3390/ijms24076768

**Published:** 2023-04-05

**Authors:** Maria Consolación Milián-Sorribes, Helena Peres, Ana Tomás-Vidal, Sara Moutinho, David S. Peñaranda, Miguel Jover-Cerdá, Aires Oliva-Teles, Silvia Martínez-Llorens

**Affiliations:** 1 Aquaculture and Biodiversity Group, Institute of Animal Science and Technology, Universitat Politècnica de València, Camino de Vera, 14, 46071 Valencia, Spain; 2 Departamento de Biologia, Faculdade de Ciências da Universidade do Porto, Rua do Campo Alegre s/n, Edifício FC4, 4169-007 Porto, Portugal; 3 CIIMAR, Centro Interdisciplinar de Investigação Marinha e Ambiental, Universidade do Porto, Terminal de Cruzeiros do Porto de Leixões, Avenida General Norton de Matos, s/n, 4450-208 Matosinhos, Portugal

**Keywords:** fish oil replacement, *Seriola dumerili*, greater amberjack, antioxidant enzymes, blood parameters, fish health

## Abstract

The present study was conducted to investigate the effects of dietary fish oil replacement with a mixture of vegetable oils and probiotic supplementation on plasma biochemical parameters, oxidative stress, and antioxidant ability of *Seriola dumerili*. Specimens with an initial weight of 175 g were used. Four feeds were formulated with 0% (FO-100), 75% (FO-25), and 100% (FO-0 and FO-0+ with the addition of *Lactobacillus* probiotics) substitution of fish oil with a mixture of linseed, sunflower, and palm oils. After 109 days, no significant differences were observed in the activity of antioxidant enzymes in the liver, foregut, and hindgut, only glucose-6-phosphate dehydrogenase activity in the liver was higher in the fish fed the FO-100 diet than in those fed the FO-0 diet. No significant differences were observed in the total, reduced, and oxidized glutathione and the oxidative stress index in the liver. In addition, lipid peroxidation in the liver and red muscle values were higher in the fish fed the FO-100 diet than in the fish fed the FO-0+ diet, however, the foregut of the fish fed the FO-100 diet presented lower values than that of the fish fed the FO replacement diet, with and without probiotics. There were significant differences in cholesterol levels in the FO-100 group; they were significantly higher than those observed with the fish diets without fish oil. To sum up, fish oil can be replaced by up to 25% with vegetable oils in diets for *Seriola dumerili* juveniles, but total fish oil substitution is not feasible because it causes poor survival. The inclusion of probiotics in the FO-0+ diet had no effects on the parameters measured.

## 1. Introduction

In recent years, a large number of studies has focused on replacing fish oil (FO) in aquafeeds using fats or oils from plant or animal sources [1]. Nevertheless, high FO substitution in aquafeeds without compromising fish performance and health remains a challenge, especially in carnivorous species. Due to their low price and high and constant market availability, vegetable oils (VO) have been traditionally presented as the main option to solve the dependence on FO for aquafeeds, further contributing to more environmentally sustainable aquafeeds [2]. Furthermore, partial FO replacement with a mixture of VO sources allows for a reduction of FO dependency in fish [3,4,5,6]. Nevertheless, some VOs are rich in n-6 and n-9 fatty acids, mainly linoleic acid (18:2n-6) and oleic acid (OA, 18:1n-9), but lack n-3 long-chain polyunsaturated fatty acids (LC-PUFA), which are essential to achieve optimal fish growth [7]. Further, lack of n-3 might trigger negative effects on feed utilization, induce inflammatory reactions, and depress oxidative capacity and, consequently, may reduce fish survival [8,9,10].

High dietary FO replacement has been linked to several side effects on fish health and stress [11,12,13] depending on the level of FO replacement, alternative oils included in the diet, dietary fatty acid profile, and fish species. An imbalance of n-3/n-6 LC-PUFAs triggers a stress response via altered eicosanoid production [9]. In addition, previous studies also reported negative effects in fish feed VOs, such as an increase in plasma cortisol levels [14] and a high hepatic triacylglycerol level [15]. Continuous cortisol secretion to the bloodstream has negative effects on fish feed intake, alters lipid and protein metabolism, and induces immunosuppression, increasing susceptibility to pathogens and infection [14,16,17].

On the other hand, the improvement of fish health through optimum nutrition is mandatory in smart aquaculture. In this sense, additives such as probiotics have been included in diets to counterbalance the negative effects caused by FO substitution, resulting in the strengthening of the immune system [18,19], nutrient digestibility [20], and increased fish stress resistance and survival [21].

FO substitution caused the alterations of the activities of digestive enzymes, antioxidant enzymes and the integrity of the mucosal barrier as well as in the intestinal microbiota, which participates and plays a key role in the recovery process [22,23,24]. Therefore, one of the strategies to palliate the alterations in intestinal health is to add a probiotic in the diet to improve the digestive process and fish health [22,25]. In aquaculture, the use of probiotics is very important as they colonize the intestinal mucosa and can displace pathogens and thus improve the health of the fish. This is an important aspect to better cope with the occurrence of diseases, especially in fish fed FO-free diets [26].

The greater amberjack (*Seriola dumerili*) is an excellent candidate for aquaculture diversification due to its higher growth as compared to other Mediterranean species and excellent sensory properties [6]. As marine fish have adapted to be fed high-lipid diets formulated with FO as the main lipid source, when dietary FO is substituted by vegetable oils, aspects such as immunological status, health, and welfare should be considered as they may be compromised [27,28,29].

Thus, this study aims to assess the effect of fish oil replacement with vegetable oils in diets for *Seriola dumerili* juveniles and its possible relation to oxidative stress alteration and health as well as to evaluate the potential benefits of *Lactobacillus brevis* and *L. buchneri* in fish fed with a complete FO replacement diet in different parameters to measure oxidative stress.

## 2. Results

### 2.1. Oxidative Status

Diet composition did not affect the oxidative stress enzyme activity in the liver and intestines except for glucose-6-phosphate dehydrogenase, where activity was higher in the liver in the FO-100 group and lower in the FO-0 group (Table 1). The dietary inclusion of probiotics in the FO-0 diet increased the glucose-6-phosphate dehydrogenase activity, which showed no differences compared to the other groups.

No differences were observed in the liver for the total, reduced, or oxidized glutathione levels and for the oxidative stress index (Table 2). On the other hand, lipid peroxidation (expressed as nmols MDA per gram of tissue) was affected by diet composition in the liver, foregut, and red muscle (Table 3). In the liver, MDA was higher in the control group (FO-100) than in the FO-0+ group, while the opposite was observed in the foregut. In red muscle, MDA levels were higher in the fish fed FO-100 diets than in those fed the FO-0 and FO-0+ diets.

### 2.2. Blood Parameters

As FO substitution increased, the cholesterol level reduced, and was significantly lower in the fish fed the diets deprived of fish oil than in the control (Table 4). No further differences were observed regarding the other parameters measured.

## 3. Discussion

Previous studies in fish reported an improvement of antioxidant defenses when the level of marine fatty acids was increased in the diet for seabream [30], and likewise, n-3 HUFA levels influenced the antioxidant capacity of juvenile black seabream, increased n-3 HUFA levels in the diet lead to increased MDA content in the liver and serum [31]. Further, fish oil replacement with vegetable oils can negatively affect lipid peroxidation and oxidative stress, which could lead to impaired membrane function and the reduction of endogenous antioxidant enzyme activity in fish [32]. For instance, in a previous study with *Nibea coibor* [33], it was observed that both SOD and CAT activities increased as the levels of fish oil substitution increased, being higher in fish fed a palm oil diet rather than a fish oil diet. Furthermore, in *Oncorhynchus mykiss* broodstock [34], SOD and GPX increased with increasing levels of fish oil substitution with vegetable oils. In the same study, liver CAT activity followed a negative relationship with FO substitution. During the last few years, measures have been sought to increase the antioxidant defense activity in different fish species. Among them, microbial feed additives showed promising effects on the antioxidant enzyme activity [35].

Nevertheless, in the present study, no significant differences in oxidative stress parameters were found despite the different dietary marine fatty acids in the diets, though a tendency to decrease the antioxidant enzyme activity as fish oil substitution increased was noticed, suggesting a detriment of oxidative defenses. This trend was statistically significant only for liver glucose-6-phosphate dehydrogenase (G6PD) activity. The major physiological role of G6PD is to produce NADPH [36], crucial for reductive biosynthesis [37] required to renew reduced glutathione (GSH), preserve the integrity of cell membrane sulfhydryl groups, and detoxify hydrogen peroxide and oxygen radicals in cells [38]. Nevertheless, the differences in G6PD were only observed in the fish fed the FO-0 diet, although the highest mortality was in the fish fed the FO-0+ diet.

MDA levels indirectly reflect the antioxidant capacity and the overall oxidative status of fish [39]. In the present study, the oxidation status was different depending on the tissue studied. Lipid peroxidation levels were higher in the FO-100 diet in the liver and red muscle than in the other tissues examined. These findings agree with observations in black sea buckthorn (*Acanthopagrus schlegelii*): increased MDA content along with an increase in dietary long-chain polyunsaturated fatty acids levels (n-3 LC-HUFAs) [31]. Previous studies demonstrated that excess n-3 LC-PUFAs in the liver are more likely to cause lipid peroxidation [40].

Comparing FO-0 with FO-0+, no significant differences were found, the CAT and SOD enzyme activities increased and the decrease in MDA concentrations in the liver in the fish fed FO-0+ may suggest a slight improvement in the antioxidant capacity of the fish. Similar results to those were found in common carp [41] in which probiotics increased CAT and SOD activity, as well as antioxidant capacity. Furthermore, in *Pangasianodon hypophthalmus* [42], they found that the addition of probiotics caused an increase in digestive enzymes and enzyme activity. However, in these studies, the difference was notable between the diets containing the probiotic and the control. In our study, the poor effect of probiotics could be due to the fact that the probiotic species were not the most suitable for *Seriola dumerili* or because the concentration of probiotics was not adequate.

The MDA contents of the liver and muscle of the fish fed with high levels of vegetable oils significantly decreased, as previously reported in other species [43,44,45], but in the case of the liver, this decrease was only observed when the probiotic was added. To the best of our knowledge, this is the first study that reports the antioxidant capacity of *Lactobacillus* as a probiotic in diets with high fish oil replacement, but the results are not conclusive as the oxidative status levels in response to the dietary inclusion of probiotics were tissue-dependent.

Probiotics in fish should produce metabolites with antioxidative abilities such as glutathione, folate, and butyrate [35]. These processes could positively influence the antioxidant capacity. However, the specific roles of each probiotic species and their effects on antioxidant capacity in fish need further study.

Blood metabolites have been widely used to provide information on the nutritional status and health of fish [46,47,48]. Hematological parameters can be considered good indicators of changes in fish. Situations of anemia, nutritional deficiencies or malnutrition affect hematocrit values [5]. To our knowledge, no previous studies assessed blood parameters in response to fish oil substitution in diets for greater amberjack. In this study, except for cholesterol, blood parameters were not affected by diet composition. Plasma cholesterol significantly decreased with the increase in vegetable oils in the diets. Similar results have been found in other species when fish oil was substituted with vegetable oils, for instance, in sea bass *D. labrax* and black sea bream *Acanthopagrus schlegeli* [49,50]. This was expected as VOs are rich in phytosterols which reduce cholesterol levels [51,52].

The hemoglobin and hematocrit values were lower than those observed in another study in the same species [53], probably due to the differences in fish weight (136 g compared to the 422 g average weight in the present experiment). This has also been noticed in other fish species, such as siluroid (*Heteropneustes fossilis*) [54], where the erythrocyte and leucocyte counts and hemoglobin concentration are higher in smaller fish and decrease as the animal grows and the hematocrit values and the mean corpuscular volumes decrease with the increase in fish weight.

In this study, plasma triglycerides levels are within the range reported for greater amberjack under good nutritional status [53]. Plasma cortisol, LDH, and glucose variations can be related to differences in diet composition and physiological changes related to stress. In the present study, no differences among treatments were observed in these parameters. This is similar to what was observed in *S. quinqueradiata* [55] juveniles fed diets where fish oil was replaced by a vegetable oil. Increases in glucose and cortisol levels normally appear as a response to chronic stress in fish. In our study, the addition of probiotics caused an increase in cortisol and a decrease in glucose in the groups without FO in the diet, therefore, probiotics had no stress-mitigating effect according to the parameters studied in the blood. This is different from the results obtained in many studies in which the addition of probiotics reduced the stress in fish [35,56,57,58,59]. The lack of differences in these plasma parameters also indicates that the fish were not suffering from stress related to diet.

The results of this study indicate that full substitution of fish oil for vegetable oils does not significantly affect the *Seriola dumerili* oxidative stress. The inclusion of a *Lactobacillus* probiotic in the diet with complete FO replacement did not have positive effects on oxidative stress, and no significant differences were found in antioxidant enzyme activity, lipid peroxidation, or hematological parameters. 

## 4. Materials and Methods

### 4.1. Fish and Experimental Diets

Greater amberjack juveniles (*Seriola dumerili*) (160 ± 5.3 g) were obtained from Futuna Blue SA (Cádiz, Spain) and moved to Universitat Politècnica de València facilities. Before starting the experiment, the animals were acclimatized to the laboratory conditions for one month and fed a standard feed (52% protein and 15% lipids).

After acclimatization, 25 fish (175 g ± 3.62 g) per tank were randomly distributed in 12 tanks, three per diet, with a capacity of 1750 L. The fish were fed until apparent satiation, twice daily (9:00 a.m.–16:00 p.m.), six days a week, for 109 days. The photoperiod was natural, and all the tanks had similar light conditions. Water temperature was maintained between 17.0 °C and 19.1 °C, salinity—around 30 ± 1 g/L^−1^, oxygen level—6.7 ± 0.04 mg/L^−1^, and pH—between 7.5 and 7.8. Ammonium, nitrite, and nitrate levels were maintained at 0.18 ± 0.07, 0.37 ± 0.05, and 93.2 ± 6.88 mg/L^−1^, respectively.

Four isoproteic (52% crude protein) and isolipidic (15% crude lipids) feeds were formulated (Table 5): a control diet (FO-100) without FO substitution, a diet with 25% of FO (FO-25), and another diet without FO inclusion (FO-0). FM provided 3% of CL in all diets. Additionally, a fourth diet was prepared (FO-0+), with the same formulation as FO-0 but adding *Lactobacillus brevis* and *L. buchneri* (5.0 × 10^7^ UCF g^−1^) with a mix of 50% of each species as probiotic bacteria.

### 4.2. Diets’ Hemical Analysis

Diets’ chemical analyses were carried out in the Animal Science Technology at Universitat Politècnica de Valencia. The fatty acid composition was analyzed by gas chromatography on a FINNIGAN FOCUS 6C chromatograph (AI 3000) after direct synthesis of methyl esters (FAME) prepared according to O’Fallon [60]. Dry matter (105 °C to constant weight), ash (by incineration at 550 °C to constant weight), fat (Ankom XT10 extraction system), and protein content (Dumas method) were determined according to the AOAC [61]. The Dumas method consists in the transformation of nitrogen into the gaseous state by calcination and its determination by thermal conductivity using a LECO CN 628 protein analyzer. All the analyses were performed in triplicate.

### 4.3. Enzyme Activity and Lipid Peroxidation Determination in Muscle, Liver, and Intestines

At the end of the experiment, liver, foregut, and hindgut samples of three fish per tank (nine fish per group) were collected and immediately frozen using liquid nitrogen and stored at –80 °C for later analysis. Muscle was also collected; red muscle was carefully dissected from white muscle, and both tissues were immediately frozen with liquid nitrogen and stored at −80 °C for later analysis.

Liver and intestine samples were diluted 1:9 and 1:4, respectively, and homogenized at pH 7.8 in an ice-cold 100 mM Tris HCl buffer containing 0.1 mM EDTA and 0.1% (*v*/*v*) Triton X-100. Homogenates were centrifuged at 30,000× *g* for 30 min at 4 °C and the resulting supernatants were separated into aliquots and stored at –80 °C for further enzyme assays. All enzyme activities were measured at 37 °C on a Multiskan GO microplate reader (Model 5111 9200; Thermo Scientific, Nanjing, China).

Glucose-6-phosphate dehydrogenase (G6PDH; EC 1.1.1.49) activities were assayed as described in [62]. Enzyme activities were determined by monitoring changes in NADH or NADP absorbance at 340 nm.

Oxidative stress enzymes were assayed as follows: superoxide dismutase (SOD; EC 1.15.1.1) activity was measured at 550 nm by the ferricytochrome C method using xanthine/xanthine oxidase as a source of superoxide radicals [63]. Catalase activity (CAT; EC 1.11.1.6) was determined according to [64] by measuring the decrease in hydrogen peroxide concentration at 240 nm. Glutathione reductase activity (GR; EC 1.6.4.2) was determined at 340 nm by measuring NADPH oxidation as described in [65]. Glutathione peroxidase activity (GPX; EC 1.11.1.9) was assayed as described. The GSSG generated by GPX was reduced according to [66]. GR and the rate of NADPH consumption were monitored at 340 nm.

One unit of SOD enzyme activity was defined as the amount of enzyme required to produce a 50% inhibition of the reduction rate of ferricytochrome C. All other enzyme activities were expressed as units (CAT) or milliunits (G6PDH, GPX, and GR) per milligram of soluble protein (specific activity). One unit of enzyme activity was defined as the amount of enzyme required to transform 1 µmol of substrate per minute under the assay conditions. Total protein concentration was determined according to [67] as described above.

Hepatic, intestinal (foregut and hindgut), and muscle (white and red) lipid peroxidation levels were determined as malondialdehyde concentration (MDA) according to [68]. A 100 μL aliquot of supernatant from the homogenate was mixed with 500 μL of a prepared solution (15% (*w*/*v*) TCA (Sigma, St. Louis, MO, USA), 0.375% (*w*/*v*) thiobarbituric acid (Sigma), 80% (*v*/*v*) HCl 0.25 N, and 0.01% (*w*/*v*) butylated hydroxytoluene (BTH) (Sigma)). The mixture was heated to 100 °C for 15 min, cooled to ambient temperature, and centrifuged at 1500 g for 10 min. The supernatant was collected and the absorbance was measured at 535 nm. Concentration was expressed as nmol MDA per g of wet tissue.

### 4.4. Glutathione and Oxidative Stress Index in the Liver

Portions of the liver were homogenized in nine volumes of an ice-cold solution containing 1.3% 5-sulphosalicylic acid (*w*/*v*) and 10 mM HCl. The homogenates were centrifuged at 14,000× *g* for 10 min at 4 °C, and the supernatants were analyzed.

Total glutathione (tGSH) and GSSG were measured following the method described in [69] and [70] with modifications. Both tGSH and GSSG analyses were carried out at 37 °C, and changes in absorbance due to the reduction of 5,5′-dithiobis (2-nitrobenzoic acid) (DTNB) were monitored at 405 nm in a Multiskan GO microplate reader. The molar extinction coefficient used for DTNB was 13,600 M^−1^ × cm^−1^. Total GSH was determined using a reaction mixture containing 133 mM phosphate buffer with 5.8 mM EDTA at pH 7.4, 0.71 mM DTNB, 0.24 mM NADPH, and 1.2 IU/mL GR. GSSG was measured using an aliquot from the solution obtained after 60 min of incubation of 100 μL of the sample with 2 μL vinylpyridine and 6 μL 1.5 M triethanolamine. The reaction mixture contained 122 mM phosphate buffer with 5.4 mM EDTA at pH 7.4, 0.71 mM DTNB, 0.24 mM NADPH, and 1.2 IU/mL^−1^ GR.

The results were calculated using standard curves of reduced glutathione (GSH) and GSSG for tGSH and GSSG measurements, respectively. The GSH level was calculated by subtracting GSSG from the tGSH values. The data are expressed as nmol per gram of tissue. The oxidative stress index (OSI) was calculated as follows: OSI (%) = 100 × (2 × GSSG/tGSH).

### 4.5. Blood Parameters

At the end of the experiment, 15 fish per experimental group were anesthetized and blood samples were collected by puncturing the caudal vein using heparinized syringes with an anticoagulant. The samples were immediately stored at 4 °C for later analysis.

The glucose, LDH, cholesterol, and triglyceride concentrations were determined by ultraviolet spectrophotometry (Ortho Clinical Diagnostics, Raritan, NJ, EUA), the cortisol concentration—by chemiluminescence. The red blood cell count was determined in a Neubauer chamber, the hematocrit percentage—using a manual microhematocrit method, and hemoglobin—by means of spectrophotometry. All the analyses were performed by the ICTIOVET S.C.P. laboratory.

### 4.6. Statistical Analysis

The data were evaluated by analysis of variance (ANOVA), following a completely randomized design. To determine significant differences between the treatments, the Bonferroni test was applied at the 5% significance level, using the statistical program Statgraphics Centurion XVII.

## Figures and Tables

**Table 1:** Antioxidant enzyme activities ^1^ in the liver, foregut, and hindgut of Mediterranean yellowtail (*Seriola dumerili*) fed the experimental diets for 109 days.

	Diets			
	FO-100	FO-25	FO-0	FO-0+
Catalase				
Liver	808 ± 97.1	695 ± 125.9	596 ± 90.4	746 ± 137.5
Foregut	65.8 ± 13.27	72.8 ± 14.07	64.0 ± 14.07	42.3 ± 15.05
Hindgut	26.6 ± 12.15	30.5 ± 14.94	37.7 ± 11.50	12.0 ± 15.30
Superoxide dismutase				
Liver	127 ± 18.8	152 ± 18.8	121 ± 18.8	141 ± 91.8
Hindgut	74.7 ± 15.75	77.5 ± 19.42	56.5 ± 15.03	39.9 ± 19.22
Glutathione reductase				
Liver	12.4 ± 0.749	12.0 ± 0.764	13.3 ± 0.746	11.6 ± 0.765
Foregut	58.6 ± 8.56	55.5 ± 8.56	42.5 ± 8.56	30.0 ± 10.48
Hindgut	24.0 ± 2.98	23.3 ± 3.70	25.2 ± 2.80	22.0 ± 4.05
Glutathione peroxidase				
Liver	50.6 ± 5.87	55.8 ± 7.66	58.2 ± 5.49	52.9 ± 7.98
Foregut	435 ± 70.1	443 ± 70.1	324 ± 70.1	289 ± 79.4
Hindgut	154 ± 22.0	144 ± 22.0	126 ± 25.4	116 ± 20.7
Glucose-6-phosphate dehydrogenase				
Liver	98.6 ± 11.89 ^a^	88.4 ± 15.35 ^ab^	54.7 ± 11.16 ^b^	93.0 ± 15.19 ^ab^
Foregut	6.42 ± 1.40	5.24 ± 1.48	6.04 ± 1.40	4.03 ± 1.71
Hindgut	2.00 ± 0.595	2.85 ± 0.557	3.36 ± 0.557	2.40 ± 0.525

^1^ Enzyme activities expressed as U/mg protein^−1^ for catalase and superoxide dismutase and mU/mg protein^−1^ for glutathione reductase, glutathione peroxidase, and glucose-6-phosphate dehydrogenase. Values represent the mean ± standard error (*n* = 9). Different superscript letters indicate significant differences between the treatments (*p* < 0.05), Bonferroni test.

**Table 2:** Total glutathione (tGSH), reduced glutathione (GSH), oxidized glutathione (GSSG) and oxidative stress index (OSI) in the liver of Mediterranean yellowtail (*Seriola dumerili*) fed the experimental diets for 109 days.

	Diets			
	FO-100	FO-25	FO-0	FO-0+
tGSH (nmol g^−1^ tissue)	1254 ± 92.8	1161 ± 84.7	936 ± 92.8	1025 ± 92.8
GSH (nmol g^−1^ tissue)	1211 ± 91.8	1125 ± 83.8	914 ± 91.8	987 ± 91.8
GSSG (nmol g^−1^ tissue)	43.3 ± 6.26	35.5 ± 5.72	22.0 ± 6.26	37.7 ± 6.26
GSH/GSSG	44.4 ± 14.4	51.9 ± 14.4	72.6 ± 14.4	27.1 ± 17.1
OSI (%)	7.09 ± 1.14	6.28 ± 1.04	4.52 ± 1.14	7.44 ± 1.14

Values represent the mean ± standard error (*n* = 9).

**Table 3:** Lipid peroxidation levels (nmols MDA/g^−1^ tissue) in the liver, foregut, hindgut, white muscle, and red muscle of Mediterranean yellowtail (*Seriola dumerili*) fed the experimental diets for 109 days.

	Diets			
	FO-100	FO-25	FO-0	FO-0+
Liver	14.2 ± 0.949 ^a^	11.8 ± 0.837 ^ab^	11.3 ± 0.837 ^ab^	10.7 ± 0.837 ^b^
Foregut	10.8 ± 1.86 ^b^	12.6 ± 2.36 ^ab^	11.7 ± 1.72 ^ab^	18.2 ± 2.44 ^a^
Hindgut	11.0 ± 1.59	12.5 ± 1.59	12.6 ± 1.50	11.4 ± 1.59
White muscle	7.74 ± 1.33	6.25 ± 1.54	7.28 ± 1.33	7.15 ± 1.33
Red muscle	8.44 ± 0.468 ^a^	7.18 ± 0.505 ^ab^	5.90 ± 0.438 ^b^	6.16 ± 0.413 ^b^

Values represent the mean ± standard error (*n* = 9). Different superscript letters indicate significant differences between the treatments (*p* < 0.05), Bonferroni test.

**Table 4:** Hematological parameters of Mediterranean yellowtail (*Seriola dumerili*) fed the experimental diets for 109 days.

	Diets			
	FO-100	FO-25	FO-0	FO-0+
Hemoglobin (g/dL)	10.1 ± 0.56	9.56 ± 0.63	9.85 ± 0.46	9.10 ± 0.50
RBC ^1^ (number × 10^6^/µL)	2.59 ± 0.38	2.46 ± 0.44	2.66 ± 0.38	2.52 ± 0.41
Hematocrit (%)	30.4 ± 6.00	32.1 ± 6.95	30.9 ± 6.08	29.6 ± 6.42
Glucose (mg/dL)	97.3 ± 14.2	129.1 ± 12.0	97.2 ± 12.0	88.4 ± 12.5
Cholesterol (mg/dL)	168.2 ± 7.40 ^a^	151.4 ± 7.40 ^ab^	128.4 ± 7.40 ^b^	124.4 ± 7.40 ^b^
Triglycerides (mg/dL)	105.8 ± 7.06	103.8 ± 6.54	107.6 ± 6.54	106.6 ± 6.54
LDH ^2^ (U/L)	16,278 ± 3241.6	10,210 ± 2873.4	7049 ± 2981.9	12,155 ± 2873.4
Cortisol (µg/dL)	10.8 ± 5.14	13.9 ± 5.70	10.4 ± 5.30	13.8 ± 5.57

^1^ Red blood cells; ^2^ lactate dehydrogenase. Values represent the mean ± standard error (*n* = 14). Different superscript letters indicate significant differences between the treatments (*p* < 0.05), Bonferroni test.

**Table 5:** Formulation (g/kg^−1^) of the experimental diets. Feed ingredients and proximate composition.

	FO-100	FO-25	FO-0 ^1^
Ingredients (g/kg)			
Fish meal	350	350	350
Wheat	100	100	100
Defatted soybean meal	185	185	185
Iberian meat meal	110	110	110
Fish oil	95	24	0
Linseed oil	–	28	38
Sunflower oil	–	21	28
Palm oil	–	22	29
Multivitamins and minerals mix ^2^	20	20	20
Analyzed composition (% dry weight)			
Dry matter (%DM)	89.3	89.7	89.4
Crude protein (%CP)	52.2	52.5	52.1
Crude lipid (CL)	14.5	14.4	14.4
Ash (%)	7.3	9.1	7.4
EPA (% total FA)	5.8	4.3	2.8
DHA (% total FA)	13	7.6	4.3

^1^ The FO-0+ diet details are not shown in the table because it had the same ingredients as FO 0 except for the addition of probiotics *Lactobacillus brevis* and *Lactobacillus buchneri* (concentration of 5 mL per each 500 g). ^2^ Vitamins and minerals mixture (g/kg^−1^): premix, 25; Hill, 10; DL-a-tocopherol, 5; ascorbic acid, 5; (PO_4_)_2_Ca_3_, 5. Pre-mixture composition: retinol acetate, 1,000,000 IU/kg^−1^; calcipherol, 500 IU/kg^−1^; DL-a-tocopherol, 10; menadione sodium bisulfite, 0.8; thiamine hydrochloride, 2.3; riboflavin, 2.3; pyridoxine hydrochloride, 15; cyanocobalamin, 25; nicotinamide, 15; pantothenic acid, 6; folic acid, 0.65; biotin, 0.07; ascorbic acid, 75; inositol, 15; betaine, 100; 12 polypeptides.

## Data Availability

Not applicable.
